# Methodological changes made to NIHR-funded randomised controlled trials: review of 100 trials

**DOI:** 10.1186/s13063-026-09760-x

**Published:** 2026-05-04

**Authors:** Andrew Mott, Ellie Fitzmaurice, Judith Watson, Catriona McDaid

**Affiliations:** York Trials Unit, University of York, York, YO10 5DD UK

**Keywords:** Amendments, Methodological changes, NIHR, Review

## Abstract

**Background:**

Previous studies have reviewed protocol amendments to trial methods in commercial studies and single centres. These studies identified that many were avoidable changes. This study aimed to assess the types of changes and rationale for changes in publicly funded randomised controlled trials undertaken in the UK.

**Methods:**

The most recent 100 published randomised controlled trials, on 23rd June 2024, in two NIHR Journals (*Health Technology Assessment* and *Efficacy and Mechanism Evaluation*), were selected for review. Data was collected on trial characteristics and the changes reported in the protocol, published reports, and trial registry entry.

**Results:**

Of the 100 included trials, 90 reported changes to the methods. A total of 846 methodological changes were recorded; 45.49% of the recorded changes occurred within the first year of study conduct. A rationale was provided for 204 of the changes with 39 unique reasons given. Frequent reasons for the changes were oversight committee recommendations, site feedback, COVID, and alignment with clinical guidance. The changes were not universally reported across the report, protocol, and registry entry and less than half were reported on the trial registry.

**Conclusions:**

The majority of NIHR-funded trials make methodological changes throughout the duration of the trial, with a large proportion being made within the first year. Consideration should be given to whether a change in the way trials are designed and planned could reduce the need for changes early on. The reporting of methodological changes also needs improvement to ensure trial documentation is consistent and up to date.

**Trial registration:**

This project was registered on the OSF registry, the details of the registration are available here: 10.17605/OSF.IO/WV2GX. Registered on July 22 2024.

## Background

During the course of a randomised controlled trial (RCT), changes may need to be made to the methods or other aspects of the trial. These changes often need regulatory approval; in the UK this is done in the form of submission of an amendment to regulatory bodies. A single amendment submission may contain multiple changes to the protocol. Protocol amendments can add substantially to the workload of regulatory bodies who must process and approve the proposed changes. For example, the UK Health Research Authority reviewed 2674 new studies in 2021/2022, in the same period they reviewed 7175 substantial amendments across their 64 ethics committees [1]. Approved amendments must then be implemented across all centres or sites participating in the trial.

There is limited data on the cost of the preparation, processing, approval, and implementation of amendments but there is evidence that it comes at a substantial cost, with estimates from 2016 suggesting that the average cost to implement an amendment in the USA ranged from US$141,000 to US$535,000 [2]. There are no estimates of the cost of amendment implementation in the UK.

Several studies have assessed changes to trial methods or trial amendments [2–5]. These studies looked at how avoidable the amendments were. Getz et al. [2, 4, 5] provided levels of avoidability (completely avoidable, somewhat avoidable, somewhat unavoidable, completely unavoidable).

Joshi [3] identified all types of amendment at a single UK site, including changes to study methods and non-methodology changes (e.g. the addition of new recruiting centres, changes of named staff). The findings showed that the most common reason for trial amendments was to increase recruitment with three methodological aspects being identified (changes to sample size, changes to follow-up timing, and changes to recruitment pathways). Additional qualitative interviews with researchers and clinical delivery staff identified that avoidable amendments in this sample of trials were often due to rushing protocol development or not considering certain groups or logistical issues.

Getz et al. [2, 4, 5] have conducted three studies assessing global commercial trials (pharmaceutical companies) across all phases of research for amendments to the protocol. In the earliest study, Getz et al. (2011) [4] found that the most common changes were to study eligibility criteria and most happened before any patients were recruited. The findings are mainly driven by phase I/II studies (64%) where protocol changes may be expected more often. They also note that other common causes of a change were new safety information, requests from regulatory authorities, and changes to manufacturing of medicinal products all of which are more likely to occur in early phase research than in later-phase RCTs. Getz et al. (2016) assessed data from 836 protocols identifying that nearly half of the amendments were avoidable [2]. Getz et al. (2024) reviewed 2188 amendments across 950 protocols [5]. They defined completely avoidable changes as flaws or inconsistencies in the protocol and somewhat avoidable as those based on poor recruitment or investigator feedback. Unavoidable changes included new data/evidence, manufacturing or regulatory changes. They noted that the proportion of unavoidable changes increased from their initial assessments in 2011 and 2016 to their most recent assessment in 2024.

These previous studies are limited to either a single institution or studies run by pharmaceutical companies and are primarily early phase studies; therefore, the findings may not be applicable to later phase publicly funded RCTs. These studies also look broadly at the number and type of amendments made but do not consider in detail the types of changes that occur within those amendments, e.g. one amendment may contain multiple changes to the protocol, some changing the methodology and others making only typographical or administrative changes.

Our previous scoping review identified that there are very few interventions aimed at improving how researchers design their research projects [6]. The aim of this study is to review the number and types of changes that have been made to trial design in a UK sample of publicly funded trials after ethical approval and the rationale for these changes. This will then be used to inform how the design and planning of trials could be improved and amendments potentially avoided, by addressing these issues earlier during planning and development of trial protocols before ethical approval and starting recruitment.

## Aims and objectives

To assess the number and type of methodological changes to protocols of recent RCTs funded by the NIHR.

1. What methodological areas are changed and how are they changed?
2. When in the trial timeline are these changes made, relative to initial ethical approval?
3. What rationale is provided for changes, if any?
4. How do protocol changes vary across different study characteristics?
5. Are changes reported consistently across trial outputs?

## Methods

### Protocol and registration

The protocol was registered on the Open Science Framework on the 22nd July 2024 [7].

### Data

Records published in the *NIHR Health Technology Assessment* and *NIHR Efficacy and Mechanism Evaluation* journals were downloaded from OpenAlex [8] on the 23rd July 2024. The records were de-duplicated by Digital Object Identifier (DOI) ahead of screening.

### Eligibility

#### Inclusion

- RCTs (including randomised feasibility studies)
- Published in the NIHR Journals Library HTA or EME journals.
- Conducted solely in the UK

#### Exclusion

- Recruitment to the trial is ongoing.
- Non-randomised studies
- Reanalyses of other RCTs

### Screening

Records were screened by two independent reviewers against the eligibility criteria until the most recent 100 eligible studies were identified. Where an eligible study had multiple records in the data set the most recent one was used. The total of 100 studies was chosen to provide a broad enough sample of trials whilst also being feasible to collect the required data for each study.

### Data collection

The full report, any relevant protocols, and trial registry entries were obtained to provide a comprehensive record of methodological changes as these may not all be reported in a single output. Data were collected using an extraction form which was piloted to ensure all relevant data were collected. An initial sample of 5 trials from each journal was independently extracted by two researchers to ensure all data are collected and consistent. The remaining data collection was completed by a single reviewer with a second reviewer checking a further 10% of records.

Data was collected relating to methodological changes but did not include changes unrelated to the methodology of the study such as typographical issues, layout, changes to staffing, addition of new recruiting centres, and addition of sub-studies, etc.

The documents were reviewed for the target data in the following order: NIHR report, trial protocol (latest available version), trial registry entry. Where dates for a change differed across documents the earliest date was taken, when only partial dates were available (e.g. July 2012) the 1st day of the month was used. For platform trials involving multiple trial protocols only the master trial protocol was used.

### Analysis

The number of screened records and included records is summarised. Summaries of the characteristics of the included studies are also provided. Data were analysed to address each of the study objectives as follows:

1. What methodological areas are changed and how are they changed?
  The number of methodological changes is summarised by the mean, standard deviation, and range. The number and proportion of changes that affect each methodological area identified are summarised, grouped by the type of change.
2. When in the trial timeline are these changes made relative to initial ethical approval?
  The length of time to amendment in number of days from initial REC approval is summarised by the mean, standard deviation, and range. A histogram of time from REC approval is presented. The proportion of changes made in the first month, 6 months, and 1 year is summarised.
3. What rationale is provided for changes, if any?
  The number and proportion of changes are presented for each different rationale provided.
4. How do protocol changes vary across different study characteristics?
  Summaries of the number of changes are provided by trial characteristics (clinical specialty, trial type, journal of publication).
5. Are changes reported consistently across trial outputs?
  The number and proportion of all identified changes reported in the NIHR report, trial registry entry, and protocol are summarised. This shows the changes reported in one, two, or all three of these outputs.

## Results

### Screening and data collection

A total of 1796 (HTA: 1689, EME: 107) records were downloaded from OpenAlex. Following deduplication, a total of 1745 were available for screening. A total of 191 records were screened to identify the sample of 100 trials. Three trials were found to have more than one record in the sample and screening continued until 100 unique trials were identified.

The 5% double extraction was utilised to ensure consistency in how data was collected. No major differences were identified between extractions. The 10% double checking identified additional changes in 5 records including additional changes not previously identified and 1 recategorisation of a change.

### Included studies

Characteristics of the included studies are summarised in Table 1. The majority of the studies were HTA funded, full trials, undertaken in hospital settings. Only 2 studies were published in the newer threaded publication model. The studies were spread across a large number of clinical specialties.

**Table 1:** Characteristics of included studies

Characteristic		Count
**Journal**	HTA	74
	EME	26
**Publication model**	Monograph	98
	Threaded publication	2
**Internal pilot**	Yes	44
	No	56
**Trial type**	Pilot	1
	Feasibility	4
	Full	95
**Setting**	Hospital	75
	Primary care	7
	Mixed	6
	Community	4
	General practice	3
	Pre-hospital emergency medicine	2
	Other	3
**Clinical specialty**^**1**^	Cardiology	4
	Dermatology	4
	Endocrinology	4
	Opthamology	4
	Neurology	6
	Urology	6
	Obstetrics and gynaecology	7
	Oncology	7
	Respiratory	7
	Orthopaedics	11
	Psychiatry	14
	Paediatrics	21
	Other	29

^1^Trials could be across multiple specialities (e.g. paediatrics and orthopaedics)

The date of initial ethics approval was available for 98 of the studies; this date was not available in any of the identified outputs or on the Health Research Authority approval summary website for 2 studies. The earliest approval date was 2000 and the most recent was 2020. The date of publication was available for all studies; the earliest was in 2021 and the most recent was in 2024. The average length of time from ethics approval to publication date was 7.01 years (SD: 2.54; median: 6.33).

### Summary of changes

Methodological changes were reported in 90 of the included studies. The mean number of changes was 8.46 (SD = 8.71, range = 0 to 42). There was a total of 846 changes reported.

**Fig. 1:**
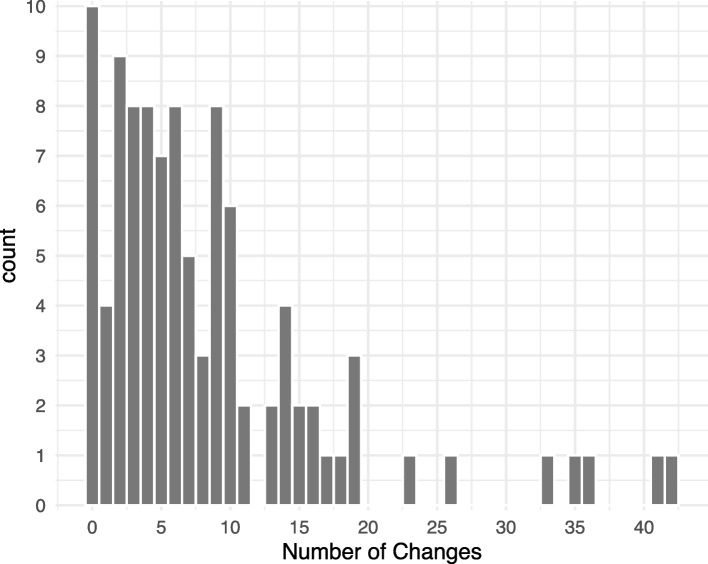
Frequency of number of methodological changes

Figure 1 is a histogram showing the frequency of the number of changes reported.

### Types of change

Each reported methodological change was categorised as below. Examples of each are provided:

#### Clarification

- Clarification of outcomes: “Clarification of text relating to the neuroimaging aspects of the protocol”—Trial ID: 22
- Clarification of eligibility criteria: “clarification that participants needed to have been off SSRIs for at least 2 weeks to meet this eligibility criterion.”—Trial ID: 181

#### Addition

- Addition of a new outcome measure: “Addition of food allergy outcome and tests”—Trial ID: 2
- Addition of new expected adverse events: “Addition of pleural effusion, venous thromboembolism and other infection to the list of expected AEs.”—Trial ID: 96

#### Reduction/removal

- Removal of an eligibility criteria: “Removal of criterion relating to latex allergy”—Trial ID: 145
- Reduction to sample size: “Sample size reduced from 600 to 510 owing to changes in trial design in relation to not including an initially planned sub-group randomisation of the conservative management arm”—Trial ID: 53

#### Correction

- Correction of analysis section: “Economic analysis section corrected to state that the contingent valuation questionnaire was for completion at baseline rather than the end of the trial”—Trial ID: 53
- Correction of outcome measure: “The version of the Medical Outcomes Study Short-Form12 (SF-12) has been changed to reflect the correct version that will be used in the study.”—Trial ID: 190

#### New method

- Change of randomisation method: “Changed from minimisation to stratification”—Trial ID: 6

#### Change (generic)*

- Change to follow-up: “enabled follow-up questionnaires to be completed by patients by post, over the telephone or online, instead of face to face.”—Trial ID: 25
- Change (generic) was also used when the nature of the change was not explicit, such as “Update to Eligibility Criteria”—Trial ID: 32

#### Summary of types of changes

The number of each type of change is summarised by area in Table 2.

**Table 2:** Type of change summarised by methodological area

Type	Eligibility	Outcomes	Analysis	Intervention	Sample size	Other	Total
Clarification	103	73	17	50	4	101	348
Change (generic)	38	42	32	35	3	75	225
Addition	49	50	5	10	5	48	167
Reduction	30	22	3	2	14	16	87
Correction	5	3	5	3	0	0	16
New method	0	0	0	0	0	3	3

The “other” methodological areas where changes were recorded were blinding, consent procedures, contamination assessment, data collection, follow-up, internal pilot, medication washout, participant procedures, placebo, randomisation, recruitment, safety reporting, screening.

### Timing of changes

**Fig. 2:**
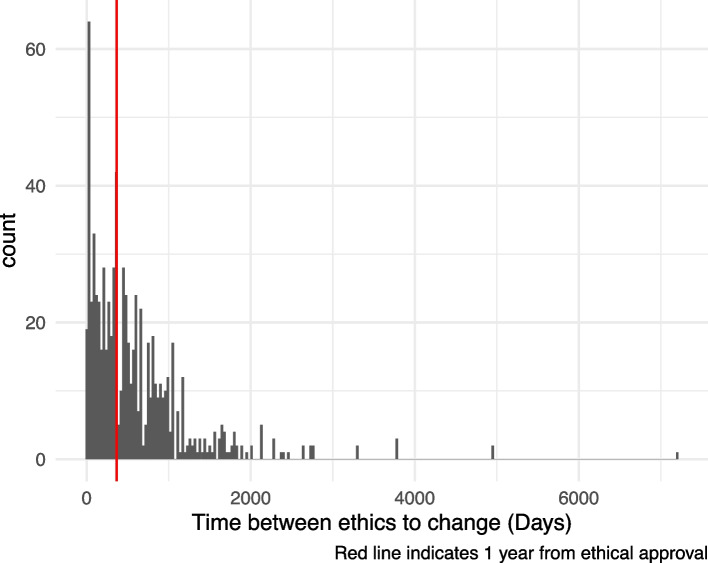
Frequency of changes by time from ethical approval

There were 754 changes where the timing of initial ethical approval and the reported timing of the change were available for analysis. The average time from ethical approval to methodological change was 599.22 days (SD: 658.98, range: 1–7202). Of the methodological changes made 45.49% (*n* = 343) were within the first year, 25.46% (*n* = 192) in the first 6 months and 5.17% (*n* = 39) in the first month. The timing of the changes is shown in Fig. 2, with 1 year from initial ethical approval marked in red.

### Rationale for changes

A rationale was provided for 204 of the 846 (24.11%) methodological changes. The rationale was categorised in each case and there were 39 unique reasons for changes. These are summarised in Table 3.

**Table 3:** Rationales provided for methodological changes

Rationale	Total^1^	Percentage of reported rationales (%)
Oversight committee recommendation	21	10.29
COVID	19	9.31
Alignment with clinical guidance	18	8.82
Site feedback	18	8.82
Align with other changes	17	8.33
Poor recruitment	15	7.35
Error	14	6.86
Funder request	10	4.90
Incorrect assumptions	9	4.41
Participant burden/feedback	9	4.41
Regulator requirement	7	3.43
Improve data collection	6	2.94
Improve retention	6	2.94
Logistics	6	2.94
New safety data	5	2.45
Reduce cost	5	2.45
Alignment with Statistical Analysis Plan	4	1.96
Analysis not possible or necessary	4	1.96
Early trial termination	4	1.96
Ensure balance in groups	4	1.96
New intervention available	4	1.96
Reduce bias	4	1.96
New outcome more appropriate to trial context	3	1.47
Improve study generalisability	2	0.98
REC request	2	0.98
Alignment with other study	1	0.49
Pilot phase findings	1	0.49

^1^Some changes provided multiple rationales

### Changes by characteristics

The number of changes (mean and SD) reported by various trial characteristics is reported in Table 4.

**Table 4:** Average number of changes by trial characteristic

Characteristic		Number of studies	Average number of changes	Standard deviation
**Trial type**	Feasibility	4	2.25	2.06
	Full	95	8.76	8.83
	Pilot	1	5.00	NA
**Journal**	EME	26	13.46	10.76
	HTA	74	6.70	7.16
**Clinical specialty**	Cardiology	4	5.50	5.74
	Colorectal	1	0.00	NA
	Dermatology	4	13.25	7.50
	Emergency	2	11.50	3.54
	Endocrinology	4	21.50	15.15
	ENT	3	10.67	8.02
	Falls	1	1.00	NA
	Fertility	1	1.00	NA
	Gastroenterology	3	10.33	5.13
	Geriatrics	1	3.00	NA
	Infectious disease	3	37.67	3.79
	Intensive care	3	8.00	3.00
	Maternity	2	2.00	2.83
	Neonatal	1	5.00	NA
	Nephrology	3	12.33	18.01
	Neurology	6	9.67	5.85
	Obstetrics and gynaecology	7	5.86	5.70
	Oncology	7	7.00	3.37
	Opthamology	4	8.00	8.16
	Orthopaedics	11	4.18	3.16
	Paediatrics	21	10.86	10.48
	Psychiatry	14	6.43	4.93
	Reproductive health	2	16.00	4.24
	Respiratory	7	7.43	5.38
	Rheumatology	1	7.00	NA
	Smoking cessation	2	7.50	3.54
	Urology	6	2.33	1.63

Full trials had more changes than feasibility or pilot trials; however, there were very few feasibility or pilot trials identified in our sample. Trials funded by EME had more changes than HTA trials. There were differences between clinical specialties but the number of trials in each case was very small.

### Reporting of changes

The reporting of each change was inconsistent across the NIHR report, trial protocol, and trial registry entry. The number and proportion of identified changes reported in each of these sources are summarised in Table 5 by methodological area. The majority of changes were reported in either the report or the trial protocol, less than half of the identified changes across all methodological areas were reported in the trial registry. Trial registries have a defined list of contents as set out by the World Health Organisation (WHO) minimum dataset [9]. Areas with a designated reporting section supplied as part of the WHO minimum dataset were better reported in the trial registry than other areas; however, these were still under 50%.

**Table 5:** Reporting of methodological changes across trial report, protocol, and trial registry

Area	Report	Protocol	Registry	Total
**Eligibility**^**1**^	171 (76%)	157 (70%)	79 (35%)	225
**Outcome**^**1**^	140 (74%)	133 (70%)	63 (33%)	190
**Intervention**^**1**^	68 (68%)	81 (81%)	16 (16%)	100
Follow-up	54 (64%)	57 (67%)	6 (7.1%)	85
Safety reporting	55 (72%)	59 (78%)	1 (1.3%)	76
Analysis	39 (63%)	42 (68%)	2 (3.2%)	62
Randomisation	20 (67%)	21 (70%)	0 (0%)	30
**Sample size**^**1**^	20 (77%)	15 (58%)	10 (38%)	26
Blinding	16 (94%)	13 (76%)	1 (5.9%)	17
Participant procedures	5 (62%)	5 (62%)	0 (0%)	8
Consent procedures	4 (67%)	4 (67%)	0 (0%)	6
Internal pilot	3 (50%)	4 (67%)	0 (0%)	6
Recruitment	5 (83%)	6 (100%)	2 (33%)	6
Medication washout	4 (100%)	2 (50%)	0 (0%)	4
Placebo	1 (50%)	1 (50%)	0 (0%)	2
Contamination assessment	1 (100%)	0 (0%)	0 (0%)	1
Data collection	1 (100%)	1 (100%)	0 (0%)	1
Screening	1 (100%)	1 (100%)	0 (0%)	1

^1^Items form part of the WHO minimum dataset

## Discussion

### Findings

This review identified 846 methodological changes, between ethical approval and study completion, amongst 100 NIHR-funded randomised controlled trials.

The majority of the reported changes occurred within the first year after ethical approval. This most probably coincides with screening and recruitment to the study commencing. The types of changes were mainly clarifications of the existing methods. Both the timing and the types of changes are potentially explained by the rationales provided with recruiting site feedback, participant feedback, logistics, and incorrect assumptions all being identified as frequent reasons. Therefore, as screening and recruitment begin, areas where the protocol may be ambiguous, unclear or logistically unfeasible are more likely to become apparent as new recruiting centres and participants join the study.

Methodological changes in order to align with clinical guidance were also common, which may not be avoidable as guidelines may change mid-trial; however, consideration of the guidelines which impact a protocol at the design stage might allow some of the changes to be avoided. COVID-19 was also a reason for many changes being implemented in these trials, being the second most commonly cited reason for change within this sample. Other frequently reported rationales were: requests from key stakeholders (oversight committees and funders); poor recruitment, and errors or incorrect assumptions.

The number of changes reported differed across different types of trials; characteristics that were associated with fewer changes included feasibility and pilot trials and HTA-funded trials. The smaller number of changes may be due to the more exploratory nature of these types of studies compared to full trials or HTA-funded trials. However, the small numbers in each group make it difficult to draw firm conclusions. The number of changes differed by clinical specialty but most specialties only had a small number of included studies.

### Comparison to other studies

Similar to our findings, Coskinas et al. [10] found that eligibility criteria were one of the most frequently changed methodological areas. We identified that the majority of changes were made within the first year of study conduct, likely when enrolment is starting. Getz et al. (2016) [2] also identified that the majority of amendments were made whilst enrolment was occurring.

Trial registry entries were poorly updated with changes to methods, even in areas where there are defined registry items for reporting certain trial methods. This has been identified in several studies related specifically to trial outcomes [11], eligibility [12], recruitment status [13], and other key characteristics [14].

Feedback from recruiting sites, participants, and oversight committees made up a large proportion of the reasons provided for why changes occurred. Involving these groups earlier in the design and setup process would allow more opportunity to clarify methods and pick up on errors. This fits with some of the findings in Joshi (2023) [3] who identified that rushed planning and lack of review of plans were key aspects identified in the qualitative interviews around the amendments made. Unlike other studies, ours did not aim to assess whether changes to the protocol were avoidable but to identify what sorts of changes were occurring so this cannot be compared.

### Strengths and limitations

This is the first study to look at the changes to RCT methods, following initial ethical approval, in publicly funded trials in the UK. Unlike previous studies, this study has explored the types of methodological changes and the reasons for the changes.

This study relied upon the reporting of these trials to identify changes; unfortunately it is clear there is significant under-reporting. For example, several of the trials where no changes were reported in the report, protocol or trial registry had multiple available versions of the protocol, yet no changes were explicitly reported as changes but these could be identified by comparing protocol versions.

How changes to methods are described also impacted the data collection for this review, which may mean there is an underestimation of the number of changes made. Some trials may describe each change individually (e.g. Inclusion criteria 4 was updated) whereas other trials described changes in broad terms (e.g. Updates were made to the eligibility criteria).

The most recent version of some protocols was often reported in the NIHR report but was not always available via the NIHR and so older versions had to be utilised for several studies.

This study specifically selected two funding schemes of the NIHR (HTA and EME); these were chosen for the large proportion of trials funded within these schemes. Trials are also funded through other NIHR schemes (e.g. RfPB, PGfAR). Trials in these other schemes are more likely to be pilot or feasibility studies which may account for the small proportion of trials identified as pilot or feasibility trials.

This sample also represents trials funded through a public funder with particular requirements, procedures and internal monitoring. These trials may therefore differ from trials with alternative funders which may not have such stringent requirements.

### Recommendations for practice

The reporting of methodological changes across trial documentation was inconsistent and there was evidence that some trials had not made clear the changes made between versions of the protocol. The CONSORT guidance for trial reporting is clear that changes should be reported but does not specify how this should be done [15]. The SPIRIT guidance for trial protocols explanation and elaboration paper makes clear that an audit trail should be available within the protocol but does not include this within the actual guidance [16]. Researchers should also ensure that processes are in place to update trial registries when changes are made to the protocol.

### Recommendations for further research

Further research should focus on:

1. Understanding the factors that lead to the need for methodological changes and whether these could be addressed in trial design and setup, prior to initial governance approvals.
2. Understanding variability in the number and types of methodological changes made across different types of trials and identifying opportunities for sharing of good practice.
3. Explore and evaluate ways to reduce the number of methodological changes that could have been incorporated within the initial trial design and planning phase.

## Conclusion

Our findings show that the majority of publicly funded randomised controlled trials have methodological changes throughout the course of the trial. Most of these changes occur within the first year of trial conduct and the reporting of these changes is inconsistent across trial sources.

## Data Availability

The data generated and analysed during the current study are available in the FigShare repository, 10.6084/m9.figshare.28730636.

## Abbreviations

CONSORT: Consolidated Standards of Reporting Trials
RCT: Randomised controlled trial
NIHR: National Institute for Health and Care Research
HTA: *Health Technology Assessment*
EME: *Efficacy and Mechanism Evaluation*
WHO: World Health Organisation
